# Comparative Analysis of Strength Improvement Techniques in Perforated Glass Fiber Reinforced Polymer Plates: Adhesive Filling, Bolt Reinforcement, and Elliptical Perforation Design

**DOI:** 10.3390/ma18184290

**Published:** 2025-09-12

**Authors:** Yiqing Dai, Jiachun Chen, Chao Yu, Ahmed D. Almutairi, Yan Yuan

**Affiliations:** 1 College of Civil Engineering, Fuzhou University, Fuzhou 350108, China; yiqing.dai@fzu.edu.cn (Y.D.);; 2 Fujian Architecture & Light-Textile Design Institute Co., Ltd., Fuzhou 350001, China; 18567907602@163.com; 3 Department of Civil Engineering, College of Engineering, Qassim University, Buraydah 51452, Saudi Arabia

**Keywords:** glass fiber reinforced polymer, stress concentration, mechanical properties, finite element analysis, bolted connection, reinforcement technique, open-hole strength

## Abstract

Due to their high strength-to-weight ratio and corrosion resistance, glass fiber reinforced polymer (GFRP) composites have been used in various civil structures. However, the GFRP profiles may be perforated to allow bolting, wiring, and pipelining, causing stress concentration and safety concerns in load-carrying scenarios. A fundamental understanding of the stress concentration mechanisms and the efficacy of mitigation techniques in such anisotropic materials remains limited, particularly for the complex stress states introduced by perforations and mechanical fasteners. This study investigates the effectiveness of three techniques, adhesive filling, bolt reinforcement, and elliptical perforation design, in mitigating stress concentration and enhancing the strength of perforated GFRP plates. The effects of perforation geometry, filler modulus, bolt types, and applied preloads on the stress concentration and bearing capacity are investigated through experimental and finite element analysis. The results reveal that steel bolt reinforcement significantly improves load-bearing capacity, achieving a 13.9% increase in the pultrusion direction and restoring nearly full strength in the transverse direction (4.91 kN vs. unperforated 4.89 kN). Adhesive filling shows limited effectiveness, with minimal load improvement, while elliptical perforations exhibit the lowest performance, reducing strength by 38% compared to circular holes. Stress concentration factors (SCF) vary with hole diameter, peaking at 5.13 for 8 mm holes in the pultrusion direction, and demonstrate distinct sensitivity to filler modulus, with optimal SCF reduction observed at 30–40 GPa. The findings highlight the anisotropic nature of GFRP, emphasizing the importance of reinforcement selection based on loading direction and structural requirements. This study provides critical insights for optimizing perforated GFRP components in modular construction and other civil engineering applications.

## 1. Introduction

Pultruded glass fiber reinforced polymer (GFRP) composites are typically composed of glass fibers embedded in a polymer resin matrix, where E-glass fibers and unsaturated polyester matrix are most commonly used [1]. The unidirectional fiber alignment provides exceptional tensile strength along the primary loading direction, while the resin matrix ensures structural integrity and facilitates the manufacturing process [2]. Both fiber orientation and resin type can be tailored to meet specific engineering requirements, offering enhanced design flexibility [3,4]. Due to the inherent properties of glass fibers and polymer resins, GFRP exhibits an outstanding strength-to-weight ratio and superior corrosion resistance against harsh environments, including exposure to acids, alkalis, moisture, and seawater [5]. These advantages have led to growing adoption in civil and structural engineering applications, such as bridge decks [4], floor panels [6], wind turbine blades [7], and reinforcement for both concrete [8,9] and soil slopes [10].

The density of GFRP typically ranges from 1700 to 2100 kg/m^3^ [11,12], representing only 20% to 25% of the density of steel (approximately 7850 kg/m^3^). This advantage in density enables GFRP to substantially reduce the overall weight of structures when used as a constructional building material [13,14]. Given this characteristic, GFRP components hold considerable promise for application in modular construction, where prefabricated elements are factory-produced and assembled on-site [15,16]. Compared with traditional cast-in-place concrete construction methods, modular construction demonstrates enhanced efficiency, shorter construction periods, and reduced dust and noise pollution. Consequently, the application and policy support for GFRP modular construction have been increasingly recognized [17,18].

GFRP components are primarily connected through adhesive bonding [19] and mechanical connections [20,21], such as bolted connections, due to the lack of weldability of GFRP materials [22,23]. Among these connection methods, bolted connections are widely utilized, particularly when considering the disassemblability and reusability of the components. It usually requires drilling to facilitate these bolted connections in prefabrication [24,25]. However, there may be instances during construction where bolts are not actually installed in the pre-drilled perforations. Concerns have been raised regarding the stress concentration and strength reduction caused by openings and bolts on GFRP specimens. Stress concentration caused by perforation is a critical contributing factor to engineering safety incidents, for example, an accident involving the failure of a crankpin journal of an ultralight airplane engine was reported in [26], where the combined contribution of the lubrication hole and undercut filet led to a stress concentration, which significantly contributed to the crack initiation and propagation, ultimately resulting in the catastrophic failure of the crankpin journal.

Studies have been conducted to investigate the changes in GFRP plate profiles with perforation, where stress concentration phenomena are exhibited, influenced by various parameters such as material properties, plate width, plate length, and load direction [27,28,29]. Different shapes of openings are known to generate distinct stress concentration behaviors, which significantly affect the residual strength and fatigue life of GFRP materials [30,31].

Finite element analysis has been performed by Xiong et al. on pultruded GFRP plates with perforations subjected to biaxial loading, while anisotropic effects were considered [32]. In this study, a stress concentration factor was introduced to analyze the impact of parameters such as perforation radius, width, plate height, and layer configuration on stress concentration. The results showed that the stress concentration factor for GFRP plates with perforations was lower in a biaxial tensile state compared to a uniaxial tensile state, due to the tensile pull-in effects at the edges of the openings along the length direction. Furthermore, the study revealed that the stress concentration factor reaches its maximum when GFRP plates with perforations are subjected to combined tensile and compressive loading, representing the most adverse condition. Additionally, it was demonstrated that composite materials experience pure tensile failure under high applied stresses, while failure at lower stress levels is primarily caused by stress concentration [33].

A review of various analytical methods for analyzing the effects of openings in composite materials has been conducted by Koord et al. [34], including analytical, experimental, and numerical approaches. Finite element methods and experiments were employed to investigate stress distribution in 0° and 90° laminated plates with openings, where width-to-diameter ratios of 3 and 5 were examined. Their findings indicated that when the width-to-diameter ratio is less than 3, the analytical solution demonstrates a high degree of accuracy. The fatigue behavior of perforated and bolted pultruded GFRP laminates is experimentally investigated in this study through a combination of static tensile and cyclic fatigue tests [35]. Digital Image Correlation (DIC) and CT techniques are employed to capture strain distribution and damage evolution around the perforations. Results highlight significant stress concentration under cyclic loading and reveal a notable “double-hole effect,” where specimens with double holes exhibit superior fatigue performance compared to single-hole configurations. Based on the experimental findings, critical geometric parameters are proposed: a diameter-to-width ratio of less than 0.3 to mitigate stress limitations in single-bolted laminates, and a critical distance-to-width ratio of 1.6 to ensure inter-hole strength and maintain consistency in design requirements. These results provide practical guidance for the design of bolted GFRP connections under fatigue loading [35,36]. A recent study systematically investigates the axial compression behavior of perforated GFRP circular tubes through experimental tests and digital image correlation analysis. The results reveal that hole size and quantity significantly reduce critical load and stiffness, while the proposed design equations demonstrate high accuracy in predicting structural performance, especially for axial stiffness [37].

A comprehensive experimental and analytical study on the performance of bolt connections in GFRP plates has been conducted, and it was found that the performance of single-bolt connections is highly dependent on the geometric dimensions of the connections [38]. The study also found that when the width-to-diameter ratio or the edge distance-to-diameter ratio of GFRP panels exceeds 5, the presence of a perforation does not significantly affect the ultimate load-bearing capacity of the plates. In bolt connections, it was noted that the bolt preload has a considerable impact on the performance of the joints. Kumar et al. [39] investigated single-bolt lap joints and concluded that a tightening torque of 15 Nm resulted in maximum strength. It was observed that, with an increase in tightening torque (contact pressure), both the load–displacement curve (stiffness) of the bolt connection and the slope of the load-bearing strength exhibited increases. Other studies [40,41] examined the fits between fasteners and perforations, revealing that gaps in the bolts can lead to a redistribution of the load between the perforations. Furthermore, recent studies have explored elliptical perforations as a means to enhance connection robustness through novel bolt designs [42].

In summary, in these studies significant attention and research have been directed to the stress concentration and strength reduction phenomena caused by openings in constructional components. Current findings indicate that the related mechanical behaviors of homogeneous materials can been analytically solved based on classical elastic mechanics, with results demonstrating good agreement with finite element methods and experimental approaches. For GFRP materials, the mechanical properties are influenced by the multilayer structure and the fiber orientation. It has been established that when the size of the openings exceeds a certain proportion relative to the profile dimensions, pronounced effects on the components occur. Moreover, the mechanical response is affected by the bolt characteristics and the preload introduced when bolts are installed in the openings.

However, despite these advancements, a fundamental scientific problem remains inadequately addressed: the lack of a systematic understanding of the stress concentration mechanisms and the efficacy of mitigation techniques in anisotropic GFRP materials with perforations and mechanical fasteners under complex stress states, which hinders the development of predictive models and design guidelines for ensuring structural reliability. To bridge this gap, this study aims to comprehensively investigate and compare the effectiveness of three reinforcement techniques (i.e., adhesive filling, bolt reinforcement, and elliptical perforation design) in mitigating stress concentration and enhancing the structural performance of perforated GFRP plates. The specific objectives include experimentally characterizing the tensile performance of perforated specimens with and without reinforcement under both loading directions, quantifying stress concentration factors and failure mechanisms using digital image correlation and finite element analysis, evaluating the influence of key parameters such as perforation geometry, filler modulus, bolt type, and preload level on load-bearing capacity and stress distribution, and ultimately establishing practical design recommendations for optimizing perforated GFRP components in modular construction and other civil engineering applications.

To mitigate the stress concentration and strength reduction potentially caused by openings and bolts in GFRP components, this study first investigates the impact of central circular openings on the stress distribution and strength of GFRP plate specimens through tensile testing conducted before and after perforation. Subsequently, tensile specimens are designed and fabricated based on adhesive filling, bolt reinforcement, and elliptical perforation design, and their effects on enhancing the strength of the perforated specimens are examined and compared. Furthermore, a finite element model of the perforated GFRP plate specimens is established based on the material testing and tensile testing results. This model is utilized to further analyze the influence of different parameters, including the elastic modulus of adhesives and bolts, the preloads applied to the bolts, and the geometric dimensions of the elliptical perforations.

## 2. Tensile Test Procedures on GFRP Plate Specimens

### 2.1. Materials and Methods

**Figure 1:**
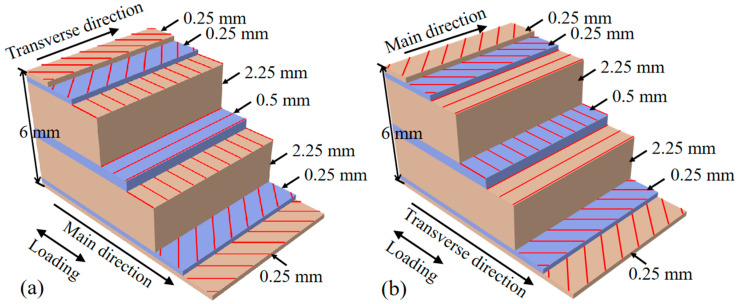
Laminate structure of GFRP plate specimens: (**a**) main fiber (pultrusion) direction; (**b**) transverse direction. (Red lines indicate fiber orientation).

To address the research objectives outlined above, an experimental program was designed to fabricate and test GFRP specimens under various reinforcement scenarios. The specimens used for the tensile and shear performance tests of GFRP plates were produced by Henan Enbeisi Composite Materials Co., Ltd. (Xinxiang, China). The GFRP plates had a thickness of 6 mm. According to the manufacture’s information, the GFRP plates were composed of E-glass fibers and unsaturated polyester matrix, and the mass fraction of fiber was 63.3%, while the volume fraction of fiber was 45.3%. As illustrated in Figure 1a,b, the plate consisted of seven layers along the thickness direction, with a fiber composition of [+45°/−45°/0°/90°/0°/+45°/−45°], and each layer of fiber had a thickness ranging from 0.25 mm to 2.25 mm. The two layers of 2.25 mm thick fiber contributed significantly to the overall fiber content; therefore, this fiber direction is referred to as the main fiber direction, which also corresponds to the pultrusion direction.

**Figure 2:**
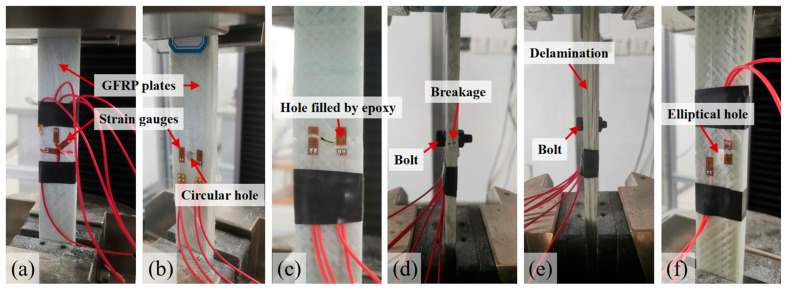
Tensile test configurations: (**a**) intact specimen; (**b**) specimen with central circular perforation; (**c**) epoxy-filled perforation; (**d**) bolted specimen under transverse loading; (**e**) bolted specimen under pultrusion-direction loading; (**f**) specimen with elliptical perforation.

The mechanical properties of the intact GFRP plate specimens, obtained from the tensile and shear pretests, are presented in Table 1. The shear performance tests of the GFRP were conducted as illustrated in Figure 2a. Following the method for 10° off-axis tensile testing referenced in the literature [43]. The elastic moduli in the pultrusion and transverse directions are denoted as *E_L_* and *E_T_*, respectively. Notably, the elastic modulus in the pultrusion direction is nearly ten times greater than that in the transverse direction, indicating a significant mechanical difference between the two directions. This difference is also reflected in the tensile strengths, denoted as *σ_L_* and *σ_T_*. The parameters *G*, *τ*, and *v* represent the shear modulus, shear strength, and Poisson’s ratio, respectively. These pronounced anisotropic properties justify the necessity of conducting tests in both the pultrusion and transverse directions to fully understand the mechanical behavior of perforated GFRP plates. These parameters were utilized in the finite element simulation of the tensile behavior of the GFRP plate specimens.

This study employs a systematic experimental approach to evaluate the tensile performance of perforated GFRP plates under various reinforcement conditions. All specimens were fabricated from pultruded GFRP plates with nominal dimensions of 250 mm × 25 mm × 6 mm and a seven-layer laminate structure (see Figure 1). The investigation is structured into tensile tests on 5 types of prepared specimens:

(1) Unperforated specimens for baseline testing to establish reference mechanical properties, comprising 6 specimens;
(2) Perforated specimens without reinforcement, comprising 6 specimens;
(3) Perforated specimens with adhesive filling, comprising 6 specimens;
(4) Perforated specimens with bolt reinforcement where three types of bolts (steel, Aluminum and Nylon) were used, comprising 18 specimens;
(5) Specimens with elliptical perforations, comprising 6 specimens.

Due to the significant differences in the mechanical properties of GFRP materials between the pultrusion and transverse directions, specimens were prepared in both orientations. The bolted specimens incorporated three types of bolts, resulting in a total of 14 distinct experimental groups. Each group consisted of three parallel specimens, leading to a total of 42 specimens.

The experimental design allows for a direct comparison of the effectiveness of each reinforcement technique in mitigating stress concentration and restoring load-bearing capacity. The following sections detail the specimen preparation and testing procedures for each group.

### 2.2. Specimens Before Perforation

The specimens were cut from pultruded GFRP plates according to the ASTM D3039/D3039M-2017 standard [44], in different orientations. The dimensions of the plate specimens were standardized at 250 mm × 25 mm × 6 mm (length × width × thickness). The tensile tests comprised two specimen groups, each consisting of three parallel specimens. One group was loaded along the main fiber direction (as shown in Figure 1a), while the other group was oriented along the transverse direction (as shown in Figure 1b). Strain gauges were positioned at the center of the plates, arranged perpendicularly in both the length and width directions., strain gauges were adhesively bonded at the center of the plate along the longitudinal and transverse directions, forming a 90° angle. Additionally, a strain gauge was affixed along the angle bisector at a 45° direction. The tensile and shear tests were performed using a universal testing machine (Instron 1185 made in the UK). The testing machine has a maximum load capacity of 100 kN and a minimum load range of 0 to 0.1 N, with a load measurement accuracy of ±0.1%. The gripping length at both ends of the plates was 5 cm, and the load was applied at 1 mm/min. The tensile loads and readouts of strain gauges were recorded at 10 Hz.

### 2.3. Specimens After Perforation with Adhesive Filling or Bolt Reinforcement

The perforated specimens for tensile testing were prepared by introducing central circular perforations in GFRP plates with dimensions of 250 mm × 25 mm × 6 mm (length × width × thickness), identical to those described in Section 2.1. The circular perforation had a diameter of 6 mm, corresponding to approximately 1/4 of the width of the specimens. As illustrated in Figure 2b, strain gauges were bonded to both sides of the circular perforation. The specimens were similarly divided into two groups, with one group oriented along the main fiber direction and the other along the transverse direction. The loading and data recording protocols were consistent with those employed for the specimens without perforation. Each group comprised three parallel specimens, resulting in a total of six specimens.

To investigate the effects of perforation filling and bolt reinforcement on alleviating stress concentration around perforations and enhancing the load-bearing capacity of the specimens, an additional 24 perforated specimens were prepared with dimensions identical to those described above. Among these, 12 specimens were aligned along the fiber direction, while the remaining 12 were oriented in the transverse direction.

Six of these specimens (equally divided between the pultrusion and transverse directions) were filled with epoxy resin to reinforce the perforations. Since epoxy resin is commonly used as the curing matrix in GFRP materials, its compatibility with the composite is excellent, including a similar coefficient of thermal expansion. The epoxy resin system consisted of E51 epoxy resin and polyamide 650 curing agent (manufactured by Yituo Composite Materials, Shanghai, China), mixed at a weight ratio of 3:1. The cured epoxy exhibited an elastic modulus of 3.2 GPa (measured based on dumb-bell specimen as per ISO 527-2 tensile testing [45,46]), closely matching the transverse modulus of the GFRP (3.76 GPa) to mitigate interfacial stress concentrations. After thorough stirring in a container, the mixture was poured into the perforations of the GFRP plates and allowed to cure for 8 h. Once cured, any excess epoxy resin on the surface was carefully removed using fine-grit sandpaper. The specimens were then post-cured for at least 7 days under ambient conditions. Prior to testing, the sanded areas were cleaned with anhydrous ethanol and degreasing cotton, followed by the attachment of strain gauges on both sides of the perforations, as illustrated in Figure 2c.

**Figure 3:**
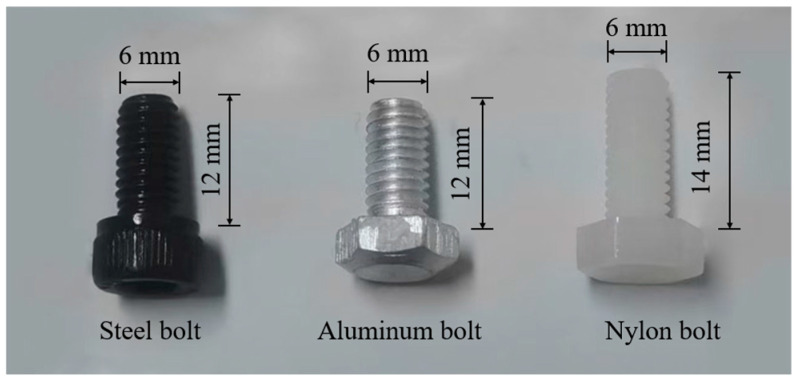
Steel, aluminum alloy and Nylon bolts used in the tests.

The remaining 18 specimens were equally distributed between the pultrusion and transverse directions, with steel, aluminum alloy, and nylon (PA66) bolts being installed, respectively, as shown in Figure 2d,e. The bolts are illustrated in Figure 3 and detailed properties are shown in Table 2 based on manufacturers’ data sheets. The 12.9-grade steel bolts, fabricated from 45# medium-carbon steel, demonstrated superior stiffness with an elastic modulus of 206 GPa. The 12.9-grade classification denotes fasteners with minimum tensile and yield strengths of 1200 MPa and 1080 MPa, respectively, representing high-performance structural components. The preloads on the bolts as listed in Table 2 were determined according to ISO 898-1:2013 [47] using a torque wrench with calibrated load cell.

### 2.4. Specimens with Elliptical Perforations

Oblong or elliptical perforations represent a strategic approach for prefabricated GFRP components, particularly in modular construction where dimensional tolerances are critical. Given the substantial size of prefabricated building elements, achieving precise perforation alignment during assembly can be challenging. The adoption of elongated openings (e.g., oval or slot-shaped perforations) accommodates minor positional deviations while ensuring successful bolt installation.

Compared to conventional circular perforations, elliptical perforations may alter stress concentration patterns and reduce load-bearing capacity. To address this knowledge gap, experimental investigations were conducted using specimens with elliptical perforations (major axis: 12 mm; minor axis: 6 mm), fabricated via abrasive waterjet cutting to ensure precision and edge integrity, as shown in Figure 2f. The specimen dimensions (250 mm × 25 mm × 6 mm) and laminate configurations were identical to those described in Section 2.2 and Section 2.3. A total of six specimens were prepared: three tested under tension along the pultrusion direction, and three along the transverse direction.

## 3. Finite Element Modeling on Specimens Under Tension

### 3.1. Specimens Before and After Perforation

To gain deeper insights into the stress distributions and failure mechanisms observed experimentally, a detailed finite element model was developed and validated against the test results. The numerical simulation of unperforated GFRP specimens was performed in ABAQUS/Standard to establish a baseline reference model. The finite element model precisely replicated the physical test specimens with nominal dimensions of 250 mm (length) × 25 mm (width) × 6 mm (thickness). The composite laminate structure, consisting of seven plies with a [+45°/−45°/0°/90°/0°/+45°/−45°] stacking sequence, was modeled using continuum shell elements (SC8R).

Material properties were implemented as an orthotropic elastic model, incorporating experimentally characterized parameters as shown in Table 1. The boundary conditions were complete constraint at one end and displacement-controlled loading at the opposite end with a total displacement of 4 mm. To ensure consistency with the experimental strain rate (1 mm/min), the analysis step duration was set to 6 s with an increment size of 0.025 s. A comprehensive mesh sensitivity analysis yielded an optimized element size of 1 mm for global regions, with refined elements of 0.2 mm in critical areas to capture stress gradients accurately. The analysis employed a static general step with geometric nonlinearity (NLGEOM) to account for potential large deformations. GFRP failure was determined based on the Tsai-Wu Failure Criterion for its ability to capture the interactive failure mechanisms between fiber-dominated and matrix-dominated failure modes in composite materials [48,49].

**Figure 4:**
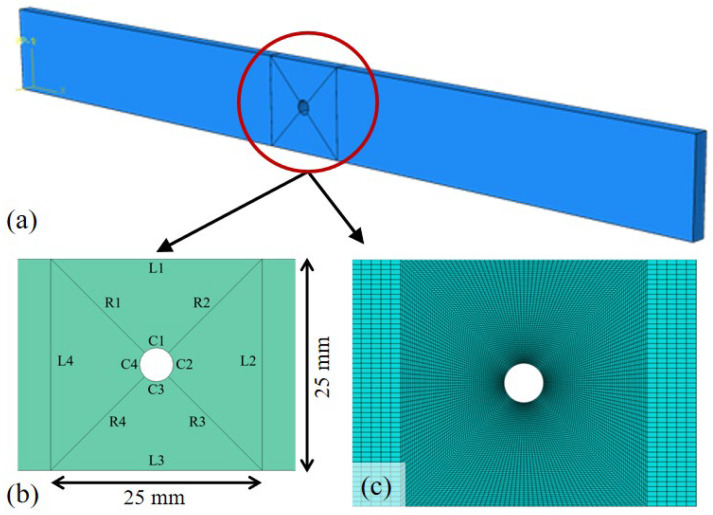
Modeling of the GFRP specimen with a central circular perforation: (**a**) full model, (**b**) partition and (**c**) meshing.

The finite element model for specimens with central circular perforations (Figure 4a) employed an identical material constitutive relationship and laminate structure as the unperforated specimens. Prior to meshing, the model was partitioned (Figure 4b) to enable localized mesh refinement around the perforation boundary, achieving a minimum element size of 0.1 mm while maintaining mesh regularity (Figure 4c). To systematically investigate the influence of perforation size on stress distribution, the diameter of circular holes was varied from 2 mm to 8 mm in the parametric study.

### 3.2. Specimens with Adhesive Filling or Bolt Reinforcement

**Figure 5:**
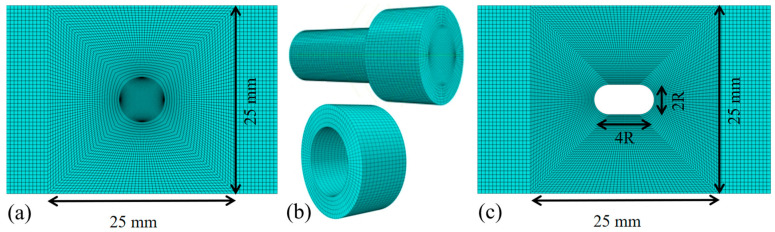
Model details for (**a**) specimens with adhesive filling, (**b**) bolts and nuts and (**c**) specimens with an elliptical perforation.

For specimen models with adhesive-filled holes, an elastic component with geometry matching the 6 mm diameter perforation was incorporated to represent the adhesive filling (Figure 5a). The adhesive’s elastic modulus was parametrically varied across 25 values ranging from 0 to 210 GPa to systematically examine its influence on mechanical performance, while maintaining a constant Poisson’s ratio of 0.38 based on typical epoxy resin properties. Considering the thin specimen geometry and potential long-term interfacial degradation, the adhesive-GFRP interaction was modeled using surface-to-surface contact with a friction coefficient of 0.4, without employing tie constraints or spring elements.

The bolt-nut assembly was modeled as shown in Figure 5b. Reflecting practical installation conditions where the bolt shank diameter is typically smaller than the hole diameter, the bolt shank was assigned a diameter of 5.8 mm with simplified geometry (threads not explicitly modeled). Contact interactions between fastener components and the GFRP specimen were defined with hard contact in the normal direction and penalty friction (μ = 0.4) in the tangential direction. Bolt-nut interfaces were constrained using tie connections. The bolt preload was applied as a concentrated force through the built-in bolt load feature in ABAQUS, maintaining a constant preload magnitude during analysis while varying values according to bolt types. Preload magnitudes were determined per ISO 898-1:2013 [47] using Equation (1):

F = A·σ(1)

where F represents the preload force in N, A is the bolt cross-sectional area in m^2^, and σ denotes the yield or failure stress of the bolt material in Pa.

### 3.3. Specimens with Elliptical Perforations

The ABAQUS model for specimens with elliptical perforations is illustrated in Figure 5c. The elliptical opening was defined with a major axis of 4R and a minor axis of 2R, with circular arcs at both ends (left and right) and straight segments along the top and bottom edges. To systematically investigate the influence of the characteristic dimension R on stress distribution, parametric studies were conducted with 2R varying from 2 mm to 8 mm in 1 mm incremental steps.

## 4. Experimental and FE Results

### 4.1. Experimental Results on Maximum Loads

In the experiments, the distinct failure modes observed, as illustrated in Figure 1a,b and Figure 2d,e, highlight the material’s anisotropic nature: transverse loading caused abrupt mid-length fractures (Figure 2d), whereas pultrusion-direction loading induced interlayer delamination (Figure 2e) rather than catastrophic rupture. This behavioral dichotomy originates from the unidirectional fiber alignment shown in Figure 1a, where transverse-direction strength relies primarily on the weaker resin matrix. The experimental results presented in Table 3 demonstrate the mechanical performance of GFRP specimens under different reinforcement conditions, with maximum loads and strains reported as mean values. For specimens loaded in the pultrusion direction, the introduction of a 6 mm circular hole (representing a 24% reduction in cross-sectional area) resulted in a 22.6% decrease in maximum load capacity, from 52.18 kN for unperforated specimens to 40.37 kN for perforated ones. A comparable reduction trend was observed in the transverse direction, where the maximum load declined by 32.7%, from 4.89 kN to 3.29 kN. These proportional reductions in load-bearing capacity align closely with the loss of cross-sectional area, suggesting that the perforation’s geometric effect dominates the initial strength reduction.

Among the tested reinforcement methods for specimens loaded in the pultrusion direction, steel bolt reinforcement demonstrated the most significant effectiveness, achieving a maximum load of 45.97 kN, which was 13.9% higher than that of unreinforced perforated specimens (40.37 kN). In contrast, other reinforcement approaches including epoxy filling, aluminum bolts, and nylon reinforcement showed minimal improvement or even slight reductions in load-bearing capacity. This performance difference may be attributed to the interlayer delamination failure mode observed during pultrusion-direction loading, as shown in Figure 2e, which is less effectively mitigated by most reinforcement methods. The high preload force applied by steel bolts appears to provide enhanced interfacial constraints between layers, thereby offering better resistance against delamination.

The presence of perforations increased the standard deviation of maximum load from 2.91 kN for unperforated specimens to 4.05 kN for perforated specimens, indicating greater variability in mechanical performance. All reinforcement methods were found to reduce this variability, suggesting they help compensate for local inconsistencies around the perforations, such as fiber misalignment or resin-rich zones caused by drilling. This stabilizing effect was particularly pronounced in steel-reinforced specimens, which exhibited a low standard deviation of 1.57 kN, likely due to the uniform stress redistribution enabled by bolt clamping.

Notably, all reinforcement techniques, with the exception of elliptical perforation design, significantly enhanced performance in the transverse loading direction. Steel bolt reinforcement demonstrated particularly effective performance, achieving a maximum load of 4.91 kN that closely approached the capacity of unperforated specimens (4.89 kN). Both hole-filling methods and bolt reinforcement effectively strengthen this critical fracture plane. This notable improvement can be attributed to the characteristic failure mode observed in transverse loading, where specimens typically fail through direct fracture across the central perforation. These reinforcements collectively contribute to restoring the structural integrity compromised by the initial perforation.

The superior performance of steel bolts is likely attributable to their combination of high stiffness and effective clamping force, which work synergistically to constrain crack propagation and redistribute stresses away from the hole boundary. This observation underscores the importance of considering both material properties and mechanical interlocking effects when designing reinforcement strategies for perforated composite structures under transverse loading conditions.

The specimens with elliptical perforations demonstrated the lowest load-bearing capacity among all tested configurations, exhibiting maximum loads of 37 kN in the pultrusion direction and 3.03 kN in the transverse direction. These values were lower than those of the unreinforced perforated specimens, suggesting that elliptical perforations may not effectively mitigate stress concentration effects or compensate for the strength reduction caused by perforations. The observed performance degradation raises potential safety concerns in structural applications.

### 4.2. Effects of Perforation Diameter

The finite element analysis of GFRP specimens with varying perforation diameters (2 to 8 mm) was conducted in ABAQUS/Explicit, incorporating the Tsai-Wu failure criterion [48,49] and progressive damage modeling [50]. The stress concentration factor (SCF), defined as the ratio of peak stress near the hole boundary to the nominal stress in the far field [51], exhibits distinct evolution patterns between transversely and pultrusion-direction loaded specimens. For transverse loading, the SCF remains relatively stable throughout the loading history until final failure, reflecting the matrix-dominated failure mechanism where stress redistribution capacity is limited. In contrast, pultrusion-direction specimens display a characteristic SCF reduction at approximately 95% of peak load, which can be attributed to the progressive damage modeling incorporating Tsai-Wu failure criterion used in the simulation. This phenomenon occurs because localized fiber fractures, while not causing immediate structural collapse, effectively redistribute stresses through the remaining intact fibers, thereby alleviating the stress concentration. The fiber-dominated damage progression allows for significant stress relief without catastrophic failure, demonstrating the material’s damage tolerance in the primary load-bearing direction.

**Figure 6:**
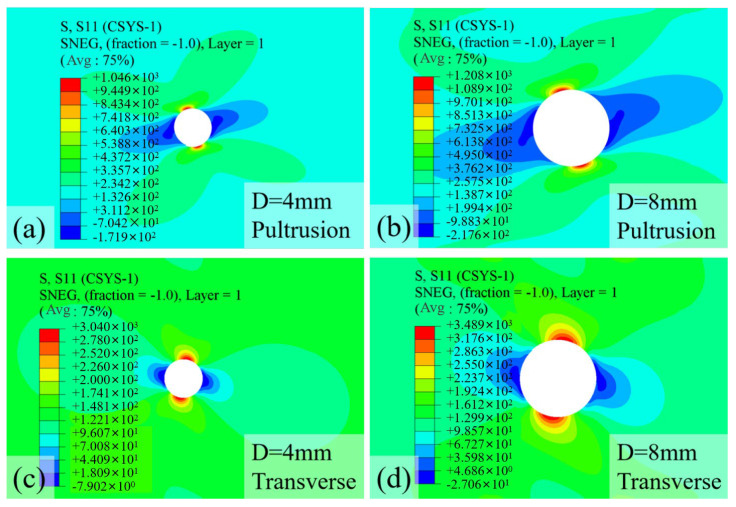
S11 stress contour plot from FE for specimens with a (**a**) 4 mm hole (pultrusion loading), (**b**) 8 mm hole (pultrusion loading), (**c**) 4 mm hole (transverse loading), and (**d**) 8 mm hole (transverse loading).

In this study, the SCF is evaluated at 90% of the maximum load as shown in Figure 6 to characterize the critical stress state prior to extensive damage accumulation. This approach provides meaningful insight into the pre-failure stress distribution while avoiding the artificial SCF reduction associated with local fiber breakage in the numerical simulation. The typical S11 stress distributions for hole diameters of 4 mm and 8 mm are presented in Figure 6, revealing that the location of maximum stress exhibits a distinct inclination angle. Notably, this inclination angle differs between transverse and pultrusion loading directions, a difference attributed to the influence of the specimen’s surface ply orientation. As illustrated in Figure 1, the outermost plies consist of ±45° fibers with a thickness of 0.25 mm, which significantly alters the stress distribution pattern compared to homogeneous materials. As shown in Figure 6a, the stress concentration zone, represented by the red regions in the contour plots, is confined to an extremely localized area around the hole perimeter. Comparative analysis of Figure 6a,b demonstrates that even with a significant increase in hole diameter from 4 mm to 8 mm, the spatial extent of the stress concentration remains remarkably limited. This highly localized nature of stress concentration, combined with its angular orientation, poses considerable challenges for experimental detection using strain gauges, as conventional measurement techniques may fail to capture these precise, rotated stress patterns.

Furthermore, examination of Figure 6c,d reveals that under transverse loading, the stress concentration zone displays a relatively larger spatial distribution. Additionally, in the region perpendicular (90°) to the maximum stress concentration, a compressive zone is observed around the hole periphery. This finding aligns with classical stress concentration theory for homogeneous materials, where such compressive zones typically exhibit lower stress magnitudes but cover more extensive areas.

**Figure 7:**
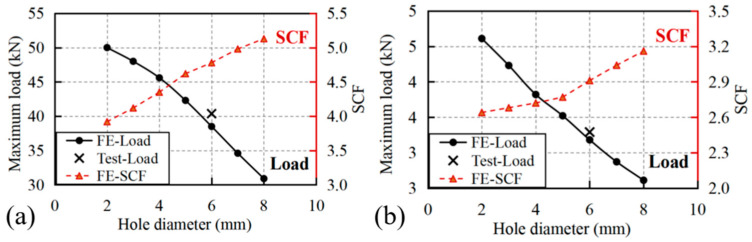
Variation in maximum loads and SCF with hole diameters for specimens loaded in (**a**) pultrusion direction and (**b**) transverse direction.

The variation in maximum loads and SCF with hole diameters is presented in Figure 7, where the simulated maximum loads exhibited strong agreement with experimental measurements at the 6 mm benchmark diameter, with deviations limited to −4.6% (pultrusion direction: predicted 38.5 kN vs. experimental 40.37 kN) and −3.3% (transverse direction: predicted 3.18 kN vs. experimental 3.29 kN). This close correlation validates the modeling approach, particularly the implementation of material property degradation laws following damage initiation.

The experimental and numerical results demonstrate distinct trends in maximum load reduction with increasing hole diameter for specimens loaded in the pultrusion and transverse directions. For pultrusion-direction loading, the maximum load exhibited a nearly linear decrease from 50 kN at 2 mm hole diameter to 30.9 kN at 8 mm diameter, representing a 38.2% reduction in load-bearing capacity. This substantial strength degradation closely corresponds to the progressive loss of cross-sectional area (24% reduction at 6 mm diameter caused a 22.6% strength decrease), indicating that the perforation’s geometric effect dominates the mechanical response. The pronounced sensitivity to hole size arises from the unidirectional fiber alignment, where larger perforations disrupt more load-bearing fibers and create severe stress concentrations in the remaining material.

In contrast, transverse-direction specimens showed a different failure pattern, with maximum loads decreasing from 4.61 kN to 2.61 kN over the same diameter range (2–8 mm). While the absolute strength reduction appears less severe than in the pultrusion direction, the relative decrease (43.4%) exceeds the cross-sectional area loss, particularly noticeable at larger hole sizes (32.7% strength reduction at 6 mm diameter versus 24% area loss). This anomalous behavior stems from the matrix-dominated failure mechanism in the transverse direction, where the weaker resin matrix is less effective at redistributing stresses around the perforation. The combined effects of reduced load path and stress concentration in the resin-rich regions lead to disproportionate strength reduction compared to the pultrusion direction, despite the lower absolute stress levels involved.

The evolution of the stress concentration factor (SCF) with increasing hole diameter exhibits distinct characteristics that reflect the anisotropic nature of GFRP laminates. As shown in Figure 7, the SCF demonstrates a progressive increase with hole diameter for both loading directions, though with markedly different trends. In the pultrusion direction, the SCF rises from 3.92 at 2 mm diameter to 5.13 at 8 mm diameter, representing a 31% increase, while the transverse direction shows a more moderate growth from 2.64 to 3.16 (20% increase) over the same diameter range. This divergence stems from the fundamental difference in load transfer mechanisms—the fiber-dominated pultrusion direction exhibits greater sensitivity to geometric discontinuities due to the direct interruption of primary load-bearing fibers, whereas the matrix-dominated transverse direction demonstrates better stress redistribution capability around larger holes. In addition, the absolute SCF values in the pultrusion direction consistently exceed those in the transverse direction by approximately 60–70%, emphasizing the critical importance of loading orientation in stress concentration analysis for composite structures.

### 4.3. Effects of Filler Modulus

**Figure 8:**
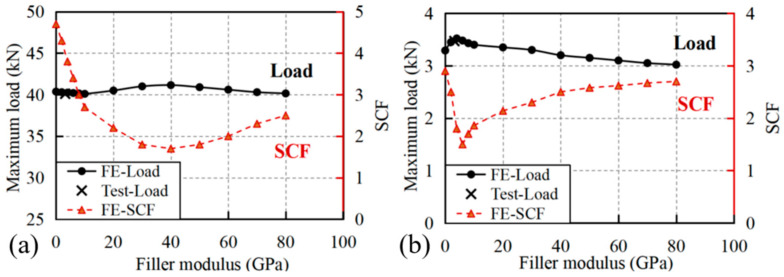
Variation in maximum loads and SCF for specimens with diffident filler moduli and loaded in (**a**) pultrusion direction and (**b**) transverse direction.

The mechanical response of perforated GFRP specimens exhibits distinct dependencies on filler modulus for both pultrusion and transverse loading directions, as shown in Figure 8. For pultrusion-direction loading, the maximum load demonstrates remarkable stability across a wide range of filler moduli (0–80 GPa), maintaining values between 40.15 kN and 41.15 kN with less than 2% variation (see Figure 8a). This insensitivity to filler stiffness suggests that the load-bearing capacity in the fiber-dominated direction is primarily governed by the continuous fiber architecture rather than the filler material properties. In contrast, the stress concentration factor (SCF) shows a more pronounced response, decreasing sharply from 4.7 to 1.7 as the filler modulus increases from 0 to 40 GPa, before gradually rising again at higher moduli.

Transverse loading specimens display a different behavior, where both maximum load and SCF show more pronounced sensitivity to filler modulus variations. The maximum load initially increases by approximately 7% (from 3.29 kN to 3.52 kN) as the filler modulus rises from 0 to 4 GPa, followed by a gradual decrease at higher moduli. This non-monotonic relationship suggests the existence of an optimal filler stiffness range (2–10 GPa) for transverse strength recovery. The SCF behavior in the transverse direction reveals a rapid initial reduction from 2.9 to 1.5 (at 6 GPa filler), followed by a steady increase to 2.7 at 80 GPa, mirroring the trend observed in pultrusion loading but with greater magnitude changes. The contrasting responses between loading directions highlight the critical influence of composite anisotropy on repair effectiveness: whereas pultrusion-direction performance is dominated by fiber continuity, transverse behavior depends more significantly on matrix-filler interactions.

The data reveals an important threshold filler modulus for both loading conditions. In the pultrusion direction, this range corresponds to the minimum SCF (1.7), while in the transverse direction it marks the transition from improving to deteriorating performance. This critical range approximately matches the transverse modulus of the GFRP itself (3.76 GPa), suggesting that modulus matching between filler and composite may optimize stress transfer. However, the benefits appear limited to SCF reduction rather than strength recovery, as maximum loads show minimal improvement even at optimal filler moduli. These findings have important implications for composite repair strategy selection. For pultrusion-dominated applications, filler modulus selection should prioritize SCF reduction (optimal 30–40 GPa), while transverse-loaded structures may benefit from lower modulus fillers (2–10 GPa) that provide modest strength recovery. The limited effectiveness of high modulus fillers (>50 GPa) in both directions challenges conventional assumptions about repair material stiffness requirements, suggesting that excessive stiffness may degrade performance by creating new stress mismatches. The non-monotonic relationship between filler modulus and bearing capacity, with an optimal range around 30–40 GPa, challenges the conventional intuition that a stiffer filler always yields better reinforcement, providing a crucial design insight specific to GFRP composites.

### 4.4. Effects of Bolt Types

**Figure 9:**
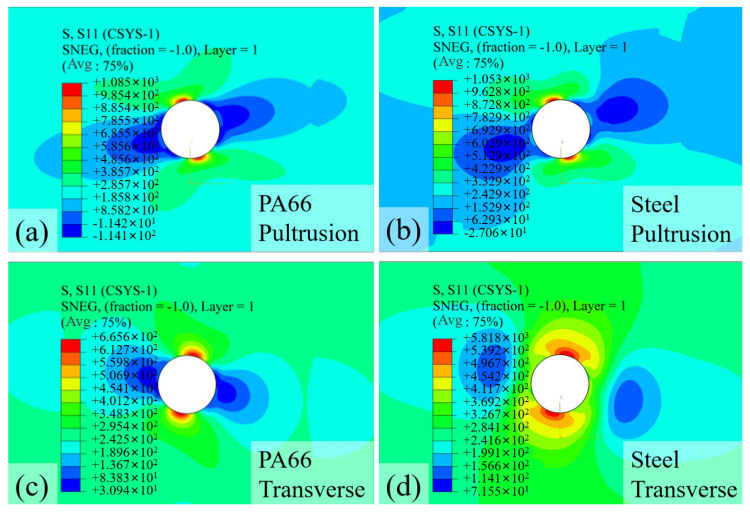
S11 stress contour plot from FE for specimens with (**a**) PA66 bolt (pultrusion loading), (**b**) steel bolt (pultrusion loading), (**c**) PA66 bolt (transverse loading), and (**d**) steel bolt (transverse loading).

Figure 9 presents the S11 stress contour plots from finite element analysis for specimens with different bolt moduli and preloads. This simulation does not represent a single-factor analysis since preloads are required to be applied according to the bolt moduli as presented in Equation (1). Comparative analysis of Figure 9a,b reveals that despite the substantial increase in bolt modulus from PA66 (8.3 GPa) to steel (206 GPa), the stress distribution patterns show relatively minor variations under pultrusion loading. The maximum stress concentration demonstrates only a modest reduction with higher modulus bolts, suggesting that bolt stiffness alone has limited influence on stress redistribution in the fiber-dominated direction. This phenomenon occurs because the primary load path remains through the continuous fibers rather than via the bolted connection.

In contrast, Figure 9c,d demonstrates different behavior under transverse loading conditions. The combination of higher modulus and greater preload in steel bolts leads to significantly enhanced reinforcement effects compared to PA66 bolts. The stress concentration zone becomes more diffuse but with lower peak values, indicating effective stress redistribution through the bolt-reinforced region. The results clearly demonstrate that bolt effectiveness depends critically on loading direction: while providing marginal benefits for pultrusion loading, high-modulus bolts substantially alter failure mechanisms in matrix-dominated transverse loading. The superior performance of steel bolts is attributed not only to their high modulus but also to the significant clamping force they generate, which suppresses delamination and improves stress redistribution through friction. This directional dependence highlights the importance of considering composite anisotropy when designing bolted connections for GFRP structures.

**Figure 10:**
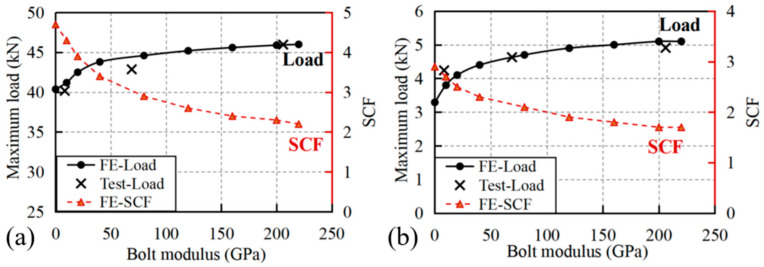
Variation in maximum loads and SCF for specimens with diffident bolts and loaded in (**a**) pultrusion direction and (**b**) transverse direction.

The variations in maximum load and stress concentration factor (SCF) with different bolt moduli are presented in Figure 10, revealing fundamental differences in reinforcement effectiveness between loading directions. For pultrusion-direction loading, the maximum load shows a moderate but consistent increase from 40.37 kN (no bolt) to 45.97 kN (steel bolt), representing a 13.9% improvement, while the SCF decreases correspondingly from 4.7 to 2.3 as shown in Figure 10a. This improvement occurs despite the relatively minor changes observed in the stress distribution patterns shown in Figure 9, suggesting that the primary reinforcement mechanism in the fiber direction involves load redistribution through the bolted region rather than significant stress concentration mitigation. The relationship between bolt modulus and performance appears nonlinear, with diminishing returns observed beyond approximately 100 GPa, indicating that excessive bolt stiffness provides limited additional benefit for pultrusion-direction loading.

For transversely loaded specimens as shown in Figure 10b, the maximum load increases by 49% (from 3.29 kN to 4.91 kN) when comparing unreinforced specimens to those with steel bolts, while the SCF decreases by 21% (from 2.9 to 2.3). This greater sensitivity to bolt properties reflects the matrix-dominated nature of transverse failure, where the bolt plays a more direct role in bridging the load path interrupted by the perforation. Interestingly, the improvement in load capacity does not correlate linearly with bolt modulus, e.g., aluminum bolts (68.9 GPa) provide nearly 90% of the strength enhancement achieved by steel bolts despite having only one-third the stiffness.

A critical observation from Figure 10 is that even the highest modulus bolts fail to fully restore the specimens’ strength to unperforated levels, particularly in the pultrusion direction where only 88% of the original capacity is recovered. This limitation highlights the fundamental challenge of replicating continuous fiber performance through discrete mechanical fasteners.

### 4.5. Effects of Elliptical Dimensions

**Figure 11:**
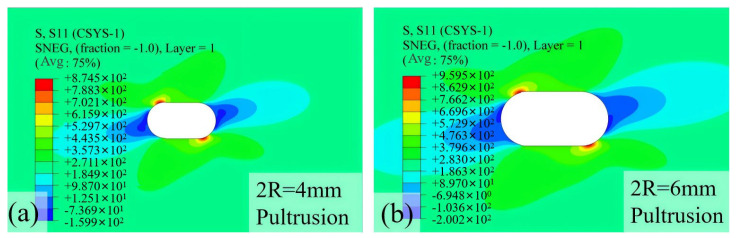
FE-simulated S11 stress distribution for pultrusion-loaded specimens with elliptical holes: (**a**) 2R = 4 mm, (**b**) 2R = 6 mm.

The FE-simulated S11 stress distributions for pultrusion-loaded specimens with elliptical perforations (2R = 4 mm and 6 mm) are presented in Figure 11, revealing consistent stress concentration patterns despite variations in hole dimensions. Both cases exhibit maximum stress concentrations at the curved ends of the elliptical holes, with the highly localized stress zones covering nearly identical areas regardless of the 2R value. However, the peak stress magnitude shows a measurable increase with larger hole dimensions, reflecting the greater geometric discontinuity introduced by the expanded perforation. Similarly to circular holes, compressive stress zones develop adjacent to the major axis ends of the ellipse, maintaining characteristic values approximately 20% of the maximum tensile stress.

**Figure 12:**
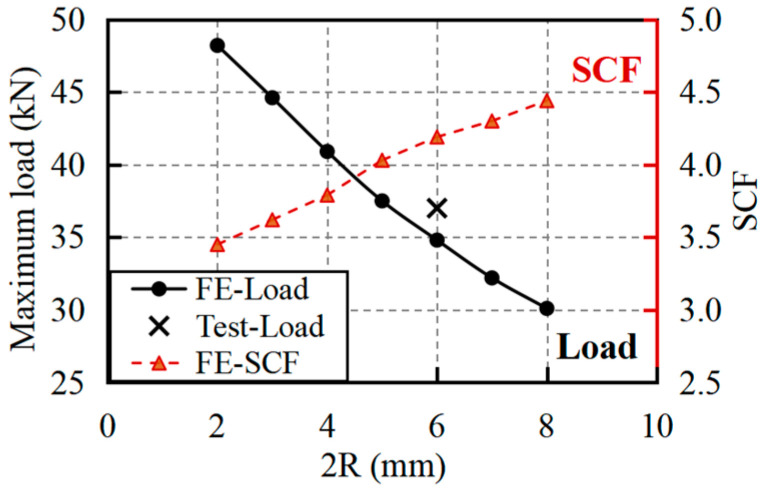
Variation in maximum loads and SCF for specimens with diffident elliptical dimensions of perforation.

Figure 12 demonstrates the variation in maximum loads and SCF with increasing elliptical dimension 2R, showing a nearly linear reduction in load capacity from 48.2 kN at 2R of 2 mm to 30.1 kN at 2R of 8 mm. This 38% strength decrease significantly exceeds the corresponding 24% reduction in net cross-sectional area, indicating that elliptical perforations introduce additional weakening mechanisms beyond simple area reduction. The SCF values follow an inverse trend, rising from 3.45 to 4.44 over the same 2R range.

The comparative analysis reveals that elliptical perforations generally produce higher SCF values than circular holes of equivalent area, particularly at larger dimensions. This performance difference stems from the elongated geometry’s tendency to create more severe stress gradients at the curved ends, despite maintaining similar compressive zones. The results highlight that while elliptical holes may offer practical advantages for assembly tolerance, they impose greater strength penalties than circular perforations, requiring careful consideration in design applications where stress concentrations govern failure. It is worth noting that, while elliptical openings have been reported in classical literature as an effective strategy for mitigating stress concentrations in isotropic materials [52], the findings of this study, based on both tensile tests and finite element analysis, suggest that this approach may not be suitable for GFRP plates.

## 5. Conclusions

This study systematically investigated the mechanical performance of perforated GFRP plates and evaluated three reinforcement techniques, i.e., adhesive filling, bolt reinforcement, and elliptical perforation design. Through experimental testing and finite element analysis, the effects of perforation geometry, filler modulus, bolt types and applied preloads on the stress concentration and bearing capacity are investigated. The main conclusions are summarized as follows:

(1) Steel bolt reinforcement provides the most effective solution, restoring load capacity to 45.97 kN in the pultrusion direction (88% of unperforated strength) and fully recovering the 4.89 kN transverse strength. This performance is achieved through significant stress concentration reduction, with SCF decreasing from 4.7 to 2.3 under optimal 15,500 N preload conditions. Therefore, high-modulus bolts provide an efficient reinforcement strategy for the design of bolted connections in GFRP modular structures.
(2) Aluminum bolts show intermediate effectiveness (42.87 kN), nylon bolts and adhesive filling exhibit limited improvements. Epoxy-filled specimens reached only 40.10 kN maximum load in pultrusion direction despite achieving optimal SCF reduction (1.7) at 30–40 GPa filler modulus.
(3) Critical relationships exist between perforation geometry and mechanical performance. Circular perforations display diameter-dependent behavior, with SCF increasing from 3.92 to 5.13 as diameter grows from 2 to 8 mm. Elliptical perforations prove particularly detrimental, showing 38% strength reduction compared to circular holes (37.00 kN vs. 40.37 kN) and elevated SCF values up to 4.44. These findings highlight the importance of geometric considerations in design applications where perforations are unavoidable, and the elliptical perforation, as a conventional stress-mitigation strategy, may not be applicable for GFRP plates.
(4) The anisotropic nature of GFRP significantly influences failure modes and reinforcement efficacy. While the pultrusion direction maintains higher absolute strength (40.37 kN vs. 3.29 kN transverse), it demonstrates greater sensitivity to stress concentration, reaching maximum SCF values of 5.13 compared to 3.16 in the transverse direction. This directional dependence underscores the need for orientation-specific design strategies in practical applications.

While this study provides valuable insights into reinforcement techniques for perforated GFRP plates, certain limitations should be acknowledged. The experimental and numerical models primarily focus on quasi-static tensile loading; thus, the long-term fatigue performance, creep behavior, and response under complex stress states (e.g., combined bending and shear) remain to be investigated. Additionally, the study considers a specific laminate configuration and hole size; further validation is needed to generalize the findings to other stacking sequences and larger-scale structural elements. Future work will focus on developing design-oriented models that integrate the effects of hole geometry, reinforcement type, and material anisotropy to predict bearing capacity and failure modes.

## Figures and Tables

**Table 1:** Mechanical properties of the GFRP specimens.

*E_L_* (MPa)	*E_T_* (MPa)	*v*	*G* (MPa)	*σ_L_* (MPa)	*σ_T_* (MPa)	τ (MPa)
36,414	3763	0.411	5000	425	42	27

**Table 2:** Mechanical properties of bolts.

Bolt Type	Material	Elastic Modulus (GPa)	Poisson’s Ratio	Preload (N)
**Steel Bolt**	45# Steel (Grade 12.9)	206.0	0.30	15,500
**Aluminum Bolt**	6061 Aluminum Alloy	68.9	0.33	3878
**Nylon Bolt**	PA66 (Nylon 66)	8.3	0.40	775

**Table 3:** Maximum loads and strains based on experimental results (parentheses indicate the standard deviation).

	Results in Pultrusion Direction		Results in Transverse Direction	
	Max Load/kN	Max Strain/%	Max Load/kN	Max Strain/%
Unperforated	52.18 (2.91)	0.92 (0.05)	4.89 (0.14)	0.83 (0.05)
Perforated	40.37 (4.05)	0.80 (0.03)	3.29 (0.13)	0.75 (0.05)
Epoxy-filled	40.10 (1.11)	0.86 (0.09)	3.47 (0.09)	0.73 (0.04)
With steel bolt	45.97 (1.57)	0.94 (0.01)	4.91 (0.19)	0.39 (0.02)
With Aluminum bolt	42.87 (1.80)	0.84 (0.07)	4.63 (0.26)	0.35 (0.02)
With Nylon bolt	40.17 (3.80)	0.75 (0.04)	4.24 (0.27)	0.39 (0.02)
Elliptical perforation	37.00 (1.00)	0.61 (0.05)	3.03 (0.23)	0.72 (0.06)

## Data Availability

The original contributions presented in this study are included in the article. Further inquiries can be directed to the corresponding author.

## References

[1] Harle S.M. (2024). Durability and long-term performance of fiber reinforced polymer (FRP) composites: A review. Structures.

[2] Zhang J., Yang J., Yu T. (2025). Durability of FRP bars and FRP-reinforced concrete beams: A critical review of accelerated aging tests and performance insights. Compos. Part B Eng..

[3] Liu Y., Su B., Zhang T. (2024). Flexural Behavior of Innovative Glass Fiber-Reinforced Composite Beams Reinforced with Gypsum-Based Composites. Polymers.

[4] Caprani C.C., Ngan J.W., Ahmadi E., Zhang S.H., Bai Y., Satasivam S. (2023). Design, construction and performance of the Monash pultruded glass fibre-reinforced polymer footbridge. Structures.

[5] Fang H., Bai Y., Liu W., Qi Y., Wang J. (2019). Connections and structural applications of fibre reinforced polymer composites for civil infrastructure in aggressive environments. Compos. Part B Eng..

[6] Almutairi A.D., Dai Y., Luo M., Bai Y. (2025). Ductile flexural behavior of composite sandwich structures with brittle fibre polymer facesheets and timber core. Compos. Struct..

[7] Li D., Fang S., Zhang Z., Wu K., Zhao J., Dai Y. (2025). Dual-Track Nonlinear Energy Sinks for Mitigating Bi-Directional Vibration of Wind Turbine Towers in Typhoons. Int. J. Struct. Stab. Dyn..

[8] Lai Z., Yan J., Wang Y., Dong C., Weng X. (2023). Axial compressive behavior and design of high-strength square concrete-filled steel tube short columns with embedded GFRP tubes. J. Constr. Steel Res..

[9] Yan J., Lai Z., Wang J., Shen Y., Zhou Z. (2025). A unified design equation to estimate the axial compressive strength of circular concrete-filled steel tube columns with embedded circular FRP tubes. J. Constr. Steel Res..

[10] Que Y., Dai Y., Hong Q., Fang L., Zhang C. (2023). Pull-Out Tests for GFRP/BFRP/Steel Bars Used as Nailing for Coal-Bearing Soil Slopes in Humid Regions. J. Test. Eval..

[11] Rafieizonooz M., Jang H., Kim J., Kim C.-S., Kim T., Wi S., Banihashemi S., Khankhaje E. (2024). Performances and properties of steel and composite prestressed tendons–A review. Heliyon.

[12] Gao H., Sun Y., Jian J., Dong Y., Liu H. (2023). Study on mechanical properties and application in communication pole line engineering of glass fiber reinforced polyurethane composites (GFRP). Case Stud. Constr. Mater..

[13] Cairoli M., Iannace G. (2024). Modular Housing Using Fibre-Reinforced Plastic Polymers (FRPs). Buildings.

[14] Liu C., Zhu R., Li F., Pan D. (2024). Load-bearing performance of a lightweight emergency bridge assembled by GFRP rectangular tubes with novel prestressed cable system. Case Stud. Constr. Mater..

[15] Bai Y. (2023). Composites for Building Assembly: Connections, Members and Structures.

[16] Zhang L., Chen K., Liu W., Liu Y., Wang K., Ge W., Guo K. (2022). Fire performance of pultruded wood-cored GFRP sandwich components for building construction. Case Stud. Constr. Mater..

[17] Zohourian M., Pamidimukkala A., Kermanshachi S., Almaskati D. (2025). Modular Construction: A Comprehensive Review. Buildings.

[18] Wdowiak-Postulak A., Świt G., Dziedzic-Jagocka I. (2024). Application of composite bars in wooden, full-scale, innovative engineering products—Experimental and numerical study. Materials.

[19] Feng P., Wu Y., Ding Y., Liu T., Tian Y. (2021). Quasi-plastic flexural behavior of adhesive-bolt hybrid connection for large scale pultruded GFRP frame. Eng. Struct..

[20] Cai Z., Bai Y., Almutairi A.D., Ding C. (2024). Low-high-low cyclic performance of screw connections for fibre-polymer composites. Constr. Build. Mater..

[21] Cai Z., Qiu C., Bai Y., Bank L.C., Zhao X.-L. (2023). Pullout behavior of connections using self-drilling screws for pultruded fiber-reinforced polymer composites in construction. J. Compos. Constr..

[22] Nhut P.V., Yoresta F.S., Duc T.Q., Matsumoto Y. (2024). Strengthening of glass fiber sheets for multi-bolted pultruded GFRP connections: Effects of connection type and bolt-tightening force. Structures.

[23] El-Naqeeb M.H., Hassanli R., Zhuge Y., Ma X., Bazli M., Manalo A. (2025). Beam-column connections in GFRP-RC moment resisting frames: A review of seismic behaviour and key parameters. Structures.

[24] Yang Z., Jia B., Sheng Y., Liu X., Zeng Y. (2025). Study on the Performance of Adhesive-Bolt Hybrid Connection Between GFRP Plate and Steel Plate. Materials.

[25] Xiang S., Cheng B., Wang J., Li D., Yan X. (2025). Behavior of hybrid bonded/bolted GFRP single-lap joint under static tensile loading: An experimental and numerical study. J. Compos. Mater..

[26] Luo Y., Yan Q., Jia B., Yu X., Zhang X., Chen Y. (2024). Study of Causes and Preventive Measures of the Support System Settlement Accident during Cast-In-Situ Bridge Construction: A Case Study. J. Bridge Eng..

[27] Nguyen-Hoang M., Becker W. (2022). Open holes in composite laminates with finite dimensions: Structural assessment by analytical methods. Arch. Appl. Mech..

[28] Qiu C., Feng P., Yang Y., Zhu L., Bai Y. (2017). Joint capacity of bonded sleeve connections for tubular fibre reinforced polymer members. Compos. Struct..

[29] Xie L., Bai Y., Qi Y., Wang H. (2019). Pultruded GFRP square hollow columns with bolted sleeve joints under eccentric compression. Compos. Part B Eng..

[30] Ekşi S., Salman L., Beşiroğlu M.F., Memişoğlu M. (2025). Open hole strength and damage behavior of GFRP and CFRP composites. Eng. Fail. Anal..

[31] Yao X., Bijlaard F., Kolstein M., Yeh H. (2005). Tensile strength and fracture behaviors of complex GFRP composites with a central hole. J. Compos. Mater..

[32] Xiong Z., Zhao C., Liu Y., Xin H., Meng Y. (2021). Biaxial stress concentration of pultruded GFRP perforated plate considering anisotropic factor. Structures.

[33] Ferdous W., Manalo A., Peauril J., Salih C., Reddy K.R., Yu P., Schubel P., Heyer T. (2020). Testing and modelling the fatigue behaviour of GFRP composites–Effect of stress level, stress concentration and frequency. Eng. Sci. Technol. Int. J..

[34] Koord J., Stüven J.-L., Petersen E., Völkerink O., Hühne C. (2020). Investigation of exact analytical solutions for circular notched composite laminates under tensile loading. Compos. Struct..

[35] Xiong Z., Meng Y., Zhao C., Liu Y., Liu X. (2024). Fatigue performance investigation of perforated and bolted pultruded glass fiber reinforced polymer laminates based on experiment and digital images. J. Compos. Mater..

[36] Xiong Z., Liu Y., Zuo Y., Xin H. (2019). Experimental evaluation of shear behavior of pultruded GFRP perforated connectors embedded in concrete. Compos. Struct..

[37] Li D., Li F. (2023). Experimental investigation on the axial compression behaviour of perforated glass fibre reinforced plastic medium-length tubes. J. Compos. Mater..

[38] Rosner C.N., Rizkalla S.H. (1995). Bolted connections for fiber-reinforced composite structural members: Analytical model and design recommendations. J. Mater. Civ. Eng..

[39] Kumar T.V., Shankar G.S., Shankar B.L. (2017). Experimental study on effect of stacking sequence, clearance and clamping torque on strength of FRP composite bolted joints. Mater. Today Proc..

[40] Zhou Y., Yazdani-Nezhad H., McCarthy M., Wan X., McCarthy C. (2014). A study of intra-laminar damage in double-lap, multi-bolt, composite joints with variable clearance using continuum damage mechanics. Compos. Struct..

[41] Liu W., Lin W. (2019). Stress around the hole of single lapped and single bolted joint plates with fitting clearance. J. Mech. Sci. Technol..

[42] Wan C., Bai Y., Ding C., Zhu L., Yang L. (2020). Mechanical performance of novel steel one-sided bolted joints in shear. J. Constr. Steel Res..

[43] Satasivam S., Satasivam S., Bai Y., Bai Y. (2016). Mechanical performance of modular FRP-steel composite beams for building construction. Mater. Struct..

[44] (2017). Standard Test Method for Tensile Properties of Polymer Matrix Composite Materials.

[45] (2025). Plastics-Determination of Tensile Properties. Part 2: Test Conditions for Moulding and Extrusion Plastics.

[46] Dai Y., Bai Y., Keller T. (2019). Stress mitigation for adhesively bonded photovoltaics with fibre reinforced polymer composites in load carrying applications. Compos. Part B Eng..

[47] (2013). Mechanical Properties of Fasteners Made of Carbon Steel and Alloy Steel. Part 1: Bolts, Screws and Studs with Specified Property Classes—Coarse Thread and Fine Pitch Thread.

[48] Xiong Z., Zhao C., Meng Y., Li W. (2024). A damage model based on Tsai–Wu criterion and size effect investigation of pultruded GFRP. Mech. Adv. Mater. Struct..

[49] Cheng X., Zhang Q., Zhang J., Guo X., Niu Z. (2019). Parameters prediction of cohesive zone model for simulating composite/adhesive delamination in hygrothermal environments. Compos. Part B Eng..

[50] Xu S., He S., Li J., Xiao B., Zhang W. (2023). A progressive damage model for quasi-static tension of 2D woven composites and FEM implementation. Compos. Struct..

[51] Mohamed H., Yang X., Shao Y., Shaheen M., Suleiman M., Zhang L., Hossian A. (2022). Stress concentration factors (SCF) of CFRP-reinforced T/Y-joints via ZPSS approach. Ocean Eng..

[52] Pilkey W.D., Pilkey D.F., Bi Z. (2020). Peterson’s Stress Concentration Factors.

